# How has the cost of antiretroviral therapy changed over the years? A database analysis in Italy

**DOI:** 10.1186/s12913-018-3507-x

**Published:** 2018-09-06

**Authors:** Lucia Taramasso, Federica Demma, Rossella Bitonti, Antonio Ferrazin, Barbara Giannini, Mauro Giacomini, Sabrina Beltramini, Elisabetta Sasso, Claudio Viscoli, Antonio Di Biagio

**Affiliations:** 1 Infectious Diseases Unit, University of Genova (DISSAL), Policlinico Hospital San Martino, Genoa, Italy; 2 Infectious Diseases Unit, Department of Internal Medicine, Fondazione IRCCS Ca’ Granda Ospedale Maggiore Policlinico, Milan, Italy; 3 EBMA Consulting, Melegnano, Milan, Italy; 4 Infectious Diseases Unit, Department of Internal Medicine, Policlinico Hospital San Martino, Genoa, Italy; 5 Department of Informatics Bioengineering, Robotics, and Systems Engineering (DIBRIS), University of Genova, Genoa, Italy; 6 Department of Pharmacy, Policlinico Hospital San Martino, Genoa, Italy

**Keywords:** HIV, AIDS, cART, Antiretroviral, Burden, Cost, Hospitalization

## Abstract

**Background:**

The number of human immunodeficiency virus (HIV)-related hospitalizations has decreased worldwide in recent years, due to the availability of combined antiretroviral therapies (cART). The present analysis aimed to analyse the economic, and clinical burden of HIV management, after the introduction of systematic use of cART.

**Methods:**

Data from HIV-infected patients, treated at Policlinico San Martino Hospital in Genova (Italy) were retrospectively collected. A comparison between years 2009 and 2015 was performed. HIV-related admissions were identified by using the Diagnosis-Related Group (DRG) codes. The resource consumption of outpatient services was derived by using a modelling approach. Expenditure for drugs was also analysed, as aggregate data.

**Results:**

The number of HIV-infected patients was 898 in 2009 and 1006 in 2015. Overall, the virological success rate improved from 2009 to 2015, as the percentage of patients with HIV-RNA < 50 copies/mL increased from 79 to 89% (*P* < 0.05). The average incidence of hospitalizations per-patient decreased from 0.30 in 2009, to 0.13 in 2015. Average expenditure per-patient decreased from €10,107 in 2009 to €9063 in 2015.

**Conclusions:**

The present analysis confirmed the role of cART in controlling HIV viral load and, consequently, in reducing hospitalizations, admissions to day-hospital and the use of outpatient services.

Clinical improvements and economic savings more than compensated the investments required to treat HIV-infected patients with cART. Health Authorities should invest in modern cART supply and universal treatment, to use at best the available resources and obtain a cost-effective improvement of health in people living with HIV. Additional research, with the involvement of different centers and the use of patient-specific data, are recommended to consolidate the findings of this analysis.

**Electronic supplementary material:**

The online version of this article (10.1186/s12913-018-3507-x) contains supplementary material, which is available to authorized users.

## Background

Recent published literature confirms that the number of persons living with human immunodeficiency virus (PLWHIV) is still rising [1]. There were approximately 36.7 million people worldwide living with human immunodeficiency virus (HIV) or acquired immune deficiency syndrome (AIDS) at the end of 2016 [1] and 130,000 living with HIV in Italy [2]. With the introduction of combined antiretroviral therapy (cART), PLWHIV have longer life expectancy and better quality of life than in the past [3]. As a consequence, HIV care is now ranking as one of the most expensive chronic diseases. Factors like: i) growth of HIV prevalence [4] ii) increase of costs of newer antiretrovirals [5]; iii) larger number of patients on chronic therapy [4, 6]; iv) guidelines suggesting early prescription of cART [7] and v) early access to treatment as prevention, are the drivers of increasing expenditure in HIV [8]. On the other side, several studies are showing that the number of HIV-related hospitalizations has decreased in recent years, primarily because of the availability of cART and, consequently, because of the declining of the incidence of advanced HIV cases [9–11]. Although antiretroviral therapy is expensive, its cost-effectiveness has been demonstrated in several analyses [12–14]. Given the opposite trend in expenditure drivers (drugs vs hospitalizations), detailed cost analyses are crucial to measure economic cost-offset. Despite several studies investigating the impact of the reduction in HIV/AIDS-related morbidity and hospitalizations in the cART era are available [8, 15, 16], breakdown of costs (i.e. between hospital, pharmacological, outpatient, etc.) has not been thoroughly characterized. This study is an attempt to achieve a better understanding of the factors affecting HIV-related hospitalizations and costs to optimize the resource allocation.

## Methods

### Study objective

To analyse the economic, clinical burden changes in HIV management, after the introduction of systematic cART prescription in last years. A comparison of the data observed in years 2009 and 2015 was performed.

### Study design

This study was an observational, retrospective analysis performed using Italian National Healthcare System (NHS) data.

### Data source

All data were collected on the MedInfo online platform enclosed in Ligurian HIV Network; (www.reteligureHIV.it). The Medinfo platform is an online database for anonymous and automatic data collection (laboratory test and clinical information) of HIV-infected patients followed in the main hospitals of Liguria (Italy) [17]. The platform is based on a web service-oriented architecture that supports the automatic and prospective transfer of laboratory data from the electronic medical records to the Ligurian HIV Network database [18]. The use of the Ligurian HIV Network database was approved by the Ligurian Ethics Committee (28 August 2013).

### Inclusion criteria

The analysis was conducted on a cohort of patients followed-up by the Infectious Disease Department of Policlinico San Martino Hospital of Genova (Italy). Patients with confirmed HIV infection diagnosis were identified as patients with at least one laboratory examination (CD4+ T cell count and HIV-RNA) per year.

### Data collection

The following resource consumption data were collected in the analysis: i) HIV-related hospitalizations / day hospital (DH); ii) outpatient services, visits and exams; iii) pharmacological treatment. HIV-related admissions were identified using the Diagnosis-Related Group (DRG) codes, 488 (HIV with extensive procedure), 489 (HIV with major related condition), 490 (HIV with or without other related condition), based on the International Classification of Diseases (ICD-9). Hospitalization data were available in aggregate form. However, for a subgroup of hospitalizations (43% of total hospitalizations in 2009 - *N* = 115 of 265- and 91% in 2015 - *N* = 117 of 129 -) a patient database, reporting individual hospital admissions, was available. A detailed analysis of such patient-level records was performed to estimate: i) severity of the disease (indicating clinical burden and considering patients’ co-morbidities and other clinical information coded during the discharge); ii) appropriateness (objective index taking into account patient characteristics, type of service provided, length of stay -LOS-, intensity of care, and level of physician involvement [19]); iii) diagnosis; iv) in-hospital mortality. A cost analysis was performed by comparing total costs between 2009 and 2015. In absence of hospital micro-costing data, hospital costs were estimating through DRG tariffs. The Italian public hospitals are remunerated for their services through fixed DRG-specific tariffs, which are set at national level and then (possibly) adapted by the Regions. The national tariffs are good proxies of standard costs sustained by the hospital (including fixed costs, such as staff and overheads, buildings, and equipment, plus variable costs, such as, patient care supplies, diagnostic and therapeutic supplies, etc.). Therefore, hospitalizations costs were estimated using the distribution of the three DRG of interest, in the subgroup of hospitalizations for which all the information was available (as per above, 43% in 2009 and 91% in 2015). The incidence values of the three DRG were then multiplied by the respective Italian tariffs [20], to calculate a weighted average hospital cost (see Additional file 1: Table S1). Similarly, the resource consumption for DH was modelled using a weighted average cost calculated by using the latest national tariffs available (2013) [20] and the number of admissions registered in Italy in years 2009 [21] and 2015 [22] (see Additional file 1: Table S1). To estimate resource consumption for outpatient services, it was assumed that patients with HIV-RNA < 50 copies/mL had 2.5 visits and exams per year (see Additional file 1: Table S2) while patients with HIV-RNA > 50 copies/mL had 4.5 visits and exams per year, according to internal hospital monitoring guidelines. The average number of visits was then multiplied by the respective Italian tariff (code 89.7; Additional file 1: Table S2) [23]. Since frequency of monitoring could vary among hospitals, to adopted clinical practice, two sensitivity analyses were run, by assuming the same number of visits and exams for all patients, regardless of viral load (Alternative Case A: 2.5 visits and exams per year; Alternative Case B: 4.5 visits and exams per year). Drug expenditure was analysed at an aggregate level. Prescriptions of treatments associated with the following ATC (Anatomical Therapeutic Chemical Classification System) codes were considered: J05AE, protease inhibitors (PI); J05AF, nucleoside and nucleotide reverse transcriptase inhibitors (NRTI); J05AG, non-nucleoside reverse transcriptase inhibitors (NNRTI); J05AR, fixed-dose combination regimens (FDC); J05AX, other antivirals (integrase, fusion and entry inhibitors). All data were processed and analysed anonymously.

### Statistical analysis

Standard descriptive statistics were used to analyse patients’ characteristics at baseline. All continuous variables were expressed as means (standard deviation) or medians (interquartile range, IQR). Categorical data were presented as percentages. For patient-level data, all tests were two-sided and a *P*-value less than 0.05 was considered statistically significant. T-test for continuous variables, chi-square test for categorical variables, z-test to compare two sample proportions for binary variables were used to detect differences between the two groups (year 2009 vs year 2015) and Mann-Whitney (rank-sum) test to compare the medians of the two groups (year 2009 vs year 2015). For aggregate data no statistical testing was conducted. Statistical analyses and calculations were performed using both Microsoft Excel 2010 (Microsoft Corporation, Redmond, WA, USA) and STATA software, release 13 (StataCorp. 2015. Stata Statistical Software: Release 13. College Station, TX: StataCorp LP).

## Results

### Patients’ characteristics

A total of *N* = 898 subjects were diagnosed with HIV infection and included in the analysis in year 2009, and *N* = 1006 in year 2015. The mean age among patients undergoing at least one hospitalization was 47 years in 2009, and 49 years in 2015. The mean CD4+ T cell count increased over the study period, from 501 cells/mm^3^ (SD: 280.55) in 2009, to 637 cells/mm^3^ (SD: 337.94) in 2015 (*P* < 0.05). The percentage of patients with optimal viral suppression (i.e. HIV-RNA persistently< 50 copies/mL) grew from 79% in 2009 to 89% in 2015 (*P* < 0.05).

### Hospitalizations (aggregate data analysis)

The average incidence of hospital admissions decreased during the observed period, from 0.30 episodes per-patient in year 2009, to 0.13 in year 2015 (*P* = 0.000). The average incidence of DH services decreased during the observed period, from 0.10 episodes per-patient in year 2009, to ≈0.00 in year 2015 (*P* = 0.000). The severity index of hospitalizations and DH increased from 1.65 in year 2009 to 1.90 in year 2015 and from 1.54 to 1.81, respectively.

### Hospitalizations (disaggregate, per-patient level analysis)

To ensure that patients’ characteristics in years 2009 and 2015 were similar, statistical tests on available data (*N* = 115 of 265 hospitalizations in 2009 and *N* = 117 of 129 in 2015) were conducted.

The proportion of male patients was not statistically different between 2009 and 2015 (69% vs 73%, *P* = 0.604). Similarly, the severity index of hospitalizations and the proportion of non-Italian patients’ nationality were not different (*P* = 0.243 and *P* = 0.245, respectively). Instead, mean age was statistically different in 2009 and 2015 (45 vs 48 years, *P* = 0.009), but perhaps such difference would be considered not relevant from an economic perspective (i.e. not plausible that resource consumption would change if age difference < 10 years, at least).

**Fig. 1:**
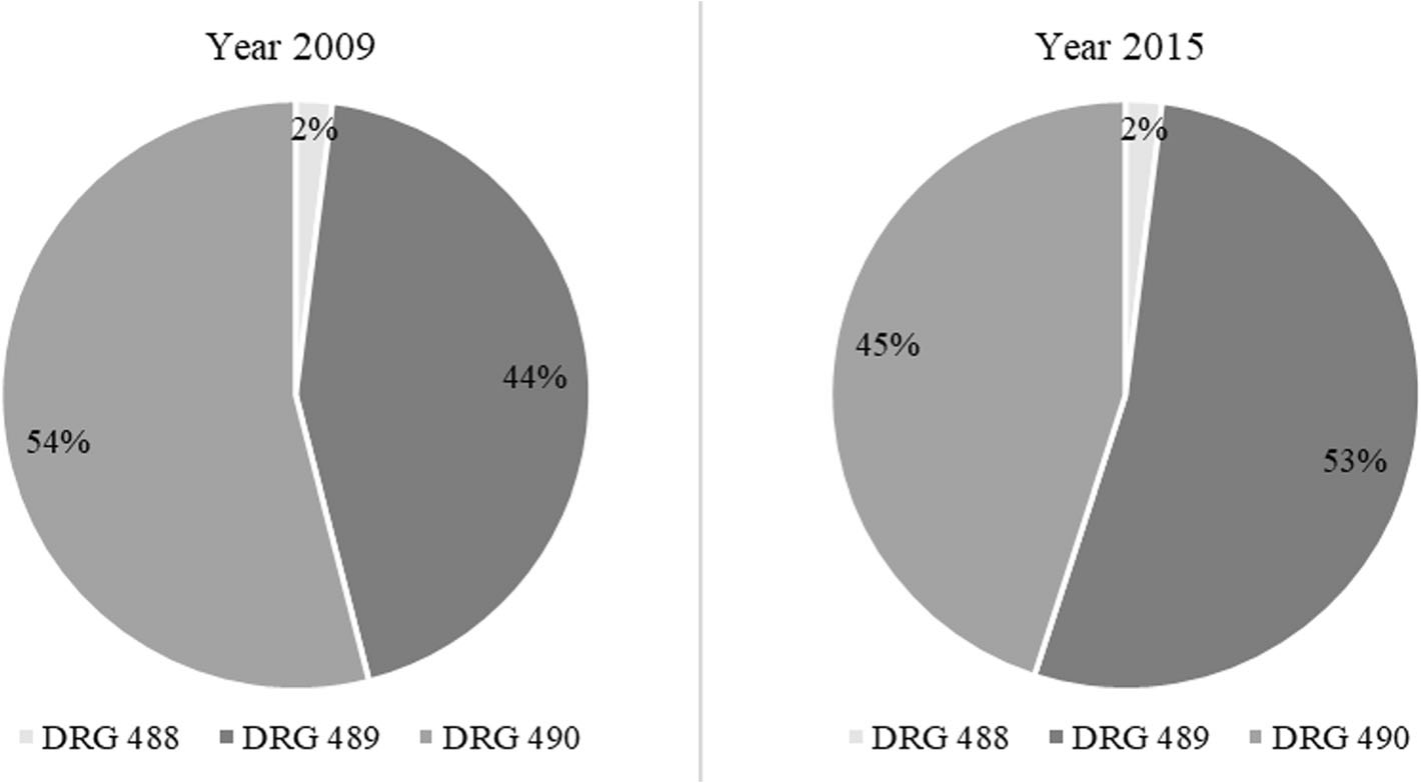
Distribution of the hospitalizations by DRG codes, (*N* = 115, Year 2009; *N* = 117, Year 2015)

A trend of increasing proportion of hospitalizations associated with the DRG code 489 (HIV with major related condition), was observed over time, but again, the difference was not statistically significant when comparing 2009 vs 2015 (*P* = 0.415; Fig. 1).

**Fig. 2:**
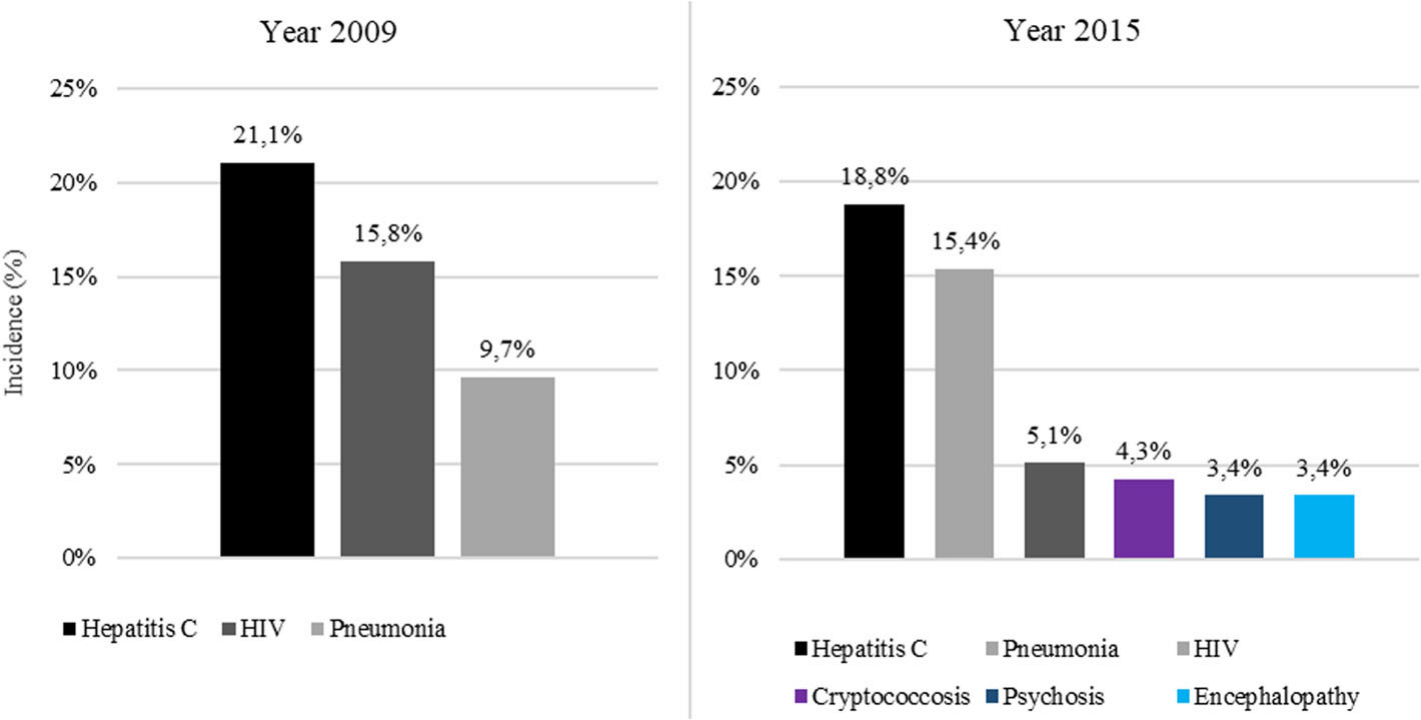
Hospitalizations by secondary diagnosis (N = 115, Year 2009; N = 117, Year 2015). HIV = human immunodeficiency virus. Only diagnoses with > 3% incidence were reported

**Fig. 3:**
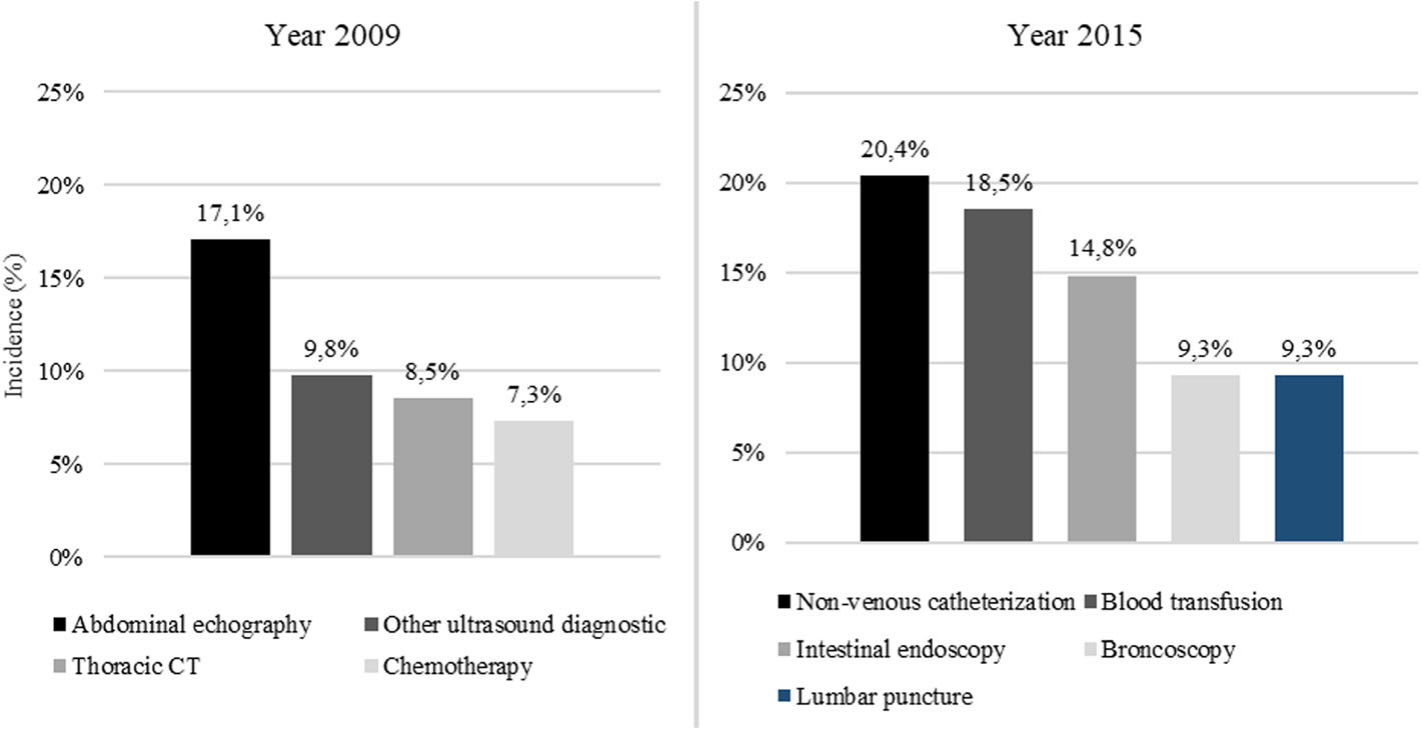
Hospitalizations by procedure (*N* = 115, Year 2009; *N* = 117, Year 2015). CT = tomography. Only procedures with > 5% incidence were reported

The proportion of appropriate hospitalizations numerically increased from 46% in 2009 to 54% in 2015 (*P* = 0.465), while the rate of pre-planned hospitalizations decreased from 10% in 2009 to 2% in 2015 (P = 0.009). The median LOS increased from 10 days (IQR: 5–16 days) in 2009 to 13 days (IQR: 7–19 days) in 2015 (*P* = 0.0331). Aetiology leading to hospitalizations was categorized as either directly or non-directly related to HIV. Hospitalizations associated with a primary diagnosis of HIV increased over the study period from 84% (2009) to 95% (2015, *P* = 0.008). A detailed analysis of secondary diagnoses and procedures performed during hospitalizations in the two study periods is shown in Figs. 2 and 3. In this analysis an increase in cryptococcosis and encephalopathy secondary diagnosis was observed over time. Moreover, procedures changed over the period of observation. In 2009 the main procedure was ultrasound diagnostic (abdominal and other side) while in 2015 procedures performed more frequently were non-venous catheterization and blood transfusion which suggest a greater severity of PLWHIV. Finally, a decreasing trend of in-hospital mortality was observed (from 6.1% in 2009 to 5.1% in 2015; *P* = 0.751).

### Costs

**Fig. 4:**
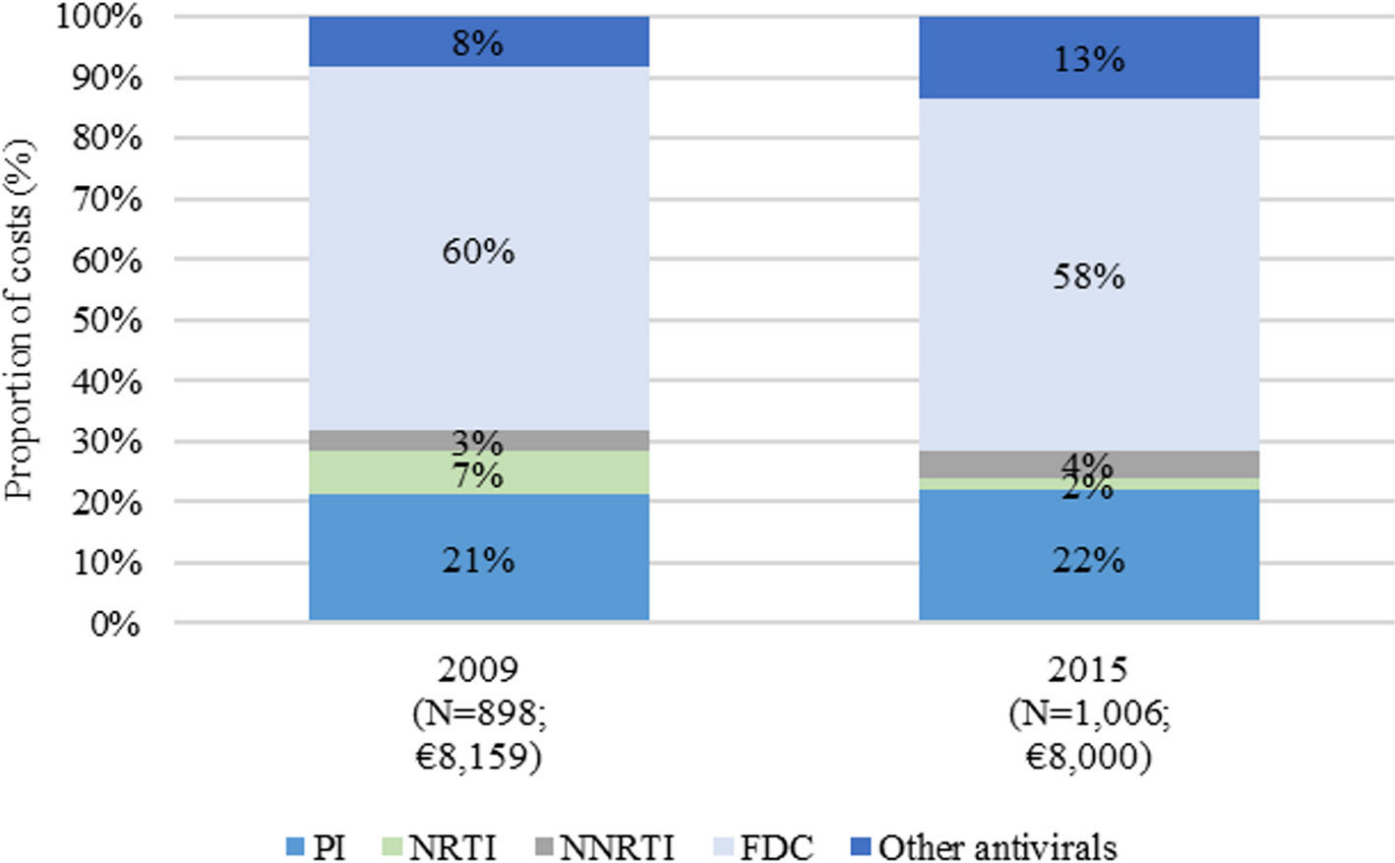
Composition of drug expenditure among ATC code J05A products. J05A = Direct Acting Antivirals; PI = protease inhibitors; NRTI = nucleoside and nucleotide reverse transcriptase inhibitors; NNRTI = non-nucleoside reverse transcriptase inhibitors; FDC = fixed-dose combination regimens

Costs of ambulatory care, exams and drugs increased from year 2009 to year 2015, whereas costs for hospitalizations and DH admissions decreased (Table 1). In the Base-case analysis the mean cost per-patient decreased from €10,107 in 2009 to €9063 in 2015, because of two factors: i) reduction of the hospitalization rate, from 29.5 to 12.8%, respectively in 2009 and in 2015 (*P* = 0.000, see Additional file 1: Table S1); ii) almost complete elimination of the day hospital service (see Additional file 1: Table S1). Figure 4 shows the distribution of pharmaceutical expenditure, in the Base-case analysis: an increase in the expenditure for “other antivirals” and a decrease in the expenditure for NRTI were basically observed over time.

**Table 1:** Results of the cost analysis

Results		Total cohort analysis		Per-patient analysis	
	Item of expenditure	Cost in Year 2009, € (*N* = 898^a^)	Cost in Year 2015, € (*N* = 1006^b^)	Cost in Year 2009, € (*N* = 898^a^)	Cost in Year 2015, € (N = 1006^b^)
Base-case analysis	Ambulatory care	51,175	54,770	57	54
	Exams	233,903	250,334	260	249
	Drugs	7,326,869	8,048,078	8159	8000
	Hospitalizations	1,439,526	763,662	1603	759
	Day-hospital access	24,924	540	28	1
	**Total per year**	**9,076,397**	**9,117,384**	**10,107**	**9063**
Sensitivity analysis (Alternative Case A)	Ambulatory care	46,382	51,960	52	52
	Exams	211,995	237,491	236	236
	Drugs	7,326,869	8,048,078	8159	8000
	Hospitalizations	1,439,526	763,662	1603	759
	Day-hospital access	24,924	540	28	1
	**Total per year**	**9,049,696**	**9,101,731**	**10,078**	**9047**
Sensitivity analysis (Alternative Case B)	Ambulatory care	83,487	93,528	93	93
	Exams	381,592	427,485	425	425
	Drugs	7,326,869	8159	1603	759
	Hospitalizations	1,439,526	1603	28	1
	Day-hospital access	24,924	28	8159	8000
	**Total per year**	**9,256,398**	**9,333,293**	**10,308**	**9278**

^a^*N* = 782 patients (87%) with HIV-RNA < 50 copies/Ml; *N* = 116 patients (13%) with HIV-RNA > 50 copies/Ml

^b^*N* = 938 patients (93%) with HIV-RNA < 50 copies/Ml; *N* = 68 patients (7%) with HIV-RNA > 50 copies/Ml

Sensitivity analyses on number of visits and exams confirmed robustness of Base-case analysis findings. In both Alternative Cases (A and B), per patients costs remained lower in the 2015 cohort compared to the 2009 cohort (absolute difference:-€1030 in both cases). Such findings confirm that monitoring costs are marginal, compared with drug costs and hospitalization costs.

## Discussion

Since 1996, the introduction of cART has improved life expectancy and quality of life of PLWHIV [24]. Nevertheless, adoption of cART at lower CD4 + T lymphocyte counts and with new and more expensive antiretrovirals has triggered increased treatment costs [25–27]. In addition, the use of highly active antiretroviral therapy (HAART) has shown greater potential of preventing HIV transmission [28]. In their study, Nosyk and Montaner have shown that the cost-effectiveness of HAART roll-out has been significantly underestimated, as economic analyses have thus far not considered the secondary benefits of HAART, chief among them the impact of HAART on HIV transmission.

The present analysis demonstrated that the increase in the expenditure for cART (from €9.08 million in 2009, to €9.10 million in 2015) was offset by a decrease in other healthcare costs, such as hospitalizations and day-hospital services. Moreover, the mean cost of cART per-patient decreased from 2009 to 2015 (from €10,107 to €9063, respectively), despite a higher use of newer drugs, such as integrase strand inhibitor and single tablet regimens.

Furthermore, this saving was accompanied by an improvement in immunological profile (higher mean CD4+ T lymphocytes counts) and HIV-RNA control (higher proportion of patients with HIV-RNA < 50 copies/ml) achieved during the study period. Indeed, an optimized use of cART, combined with a stricter regional policy for the containment of healthcare services [29], might have determined the lower incidence of hospital admissions observed in 2015 compared to 2009 and consequently a decreasing of total expenditure per patient.

Importantly, although the severity of the patients rose in 2015, the in-hospital mortality decreased from 6.1% in 2009 to 5.1% in 2015. Thus, not only the reduction of the overall costs for patient in year 2015 was achieved, but also all the patients related outcomes examined in this study improved.

The improvement of clinical outcomes and the reduction in health care consumption more than compensated the high expenditure required for cART.

In this analysis the benefit of the introduction of direct-acting antiviral agents (DAA) is not yet evident. In 2016 a new therapy era where HCV (hepatitis C virus) can be cured has begun, with remarkable results also in real-life settings and in special populations such as HCV/HIV-co-infected patients [30–32].

Furthermore, the increasing in cryptococcosis and encephalopathy, observed in our study, are a distinctive condition of a late presentation of HIV [33–35]. After many years since the beginning of the HIV epidemics, still 15–38% of patients with HIV infection continue to be diagnosed late. Late presenters demonstrate a less favorable clinical outcome and they produce a considerable and unnecessary burden for the healthcare system [36, 37].

This study has a few limitations, the most important of which concerns the “mixed” approach followed for the analysis. Aggregate data were partially used in the present analysis, while a patient-based method should be preferred, ideally. A comprehensive patient-based method should have even allowed to conduct cost analyses stratified by immunological profile/virologic control or adjusted analyses. The availability of a limited proportion of disaggregate, patient-level data for year 2009 could have influenced the significance of some results and their representativity. Furthermore, a general change in health policies in last years, aimed at containing costs, especially during the period of economic crisis, might have played a role in the changing of type and number of hospitalizations that we found in our study. Moreover, some costs were derived from assumptions (i.e. ambulatory cares and hospitalizations/DH) and this could have introduced some form of variability. Nevertheless, we do not believe these factors have substantially biased the final direction of the results, which remain valid. Finally, we believe that such results are representative of the Italian context but differences among systems of reimbursement and tariffs applied by health care services worldwide limit the applicability of the conclusions obtained by this study to other international experiences. Apart from the above-mentioned limitations, the results of the present analysis provided a pharmacoeconomic view on how cART use can impact both the clinical and economic burden of HIV. cART has transformed the management of PLWHIV over the past quarter century. However, the next years are crucial, the introduction of new drugs (TAF, new single tablet regimen INSTI based, protease inhibitor STR) will help to further improve antiretroviral treatment.

## Conclusion

The present analysis confirmed the role of cART in controlling HIV in a more effective way and, consequently, in reducing the burden of patients requiring hospitalization, admission to day-hospital and outpatient services. The gain in clinical outcomes and the reduction in health care consumption more than compensate the high expenditure required for cART. Health Authorities should invest in modern cART supply and universal treatment, to use at best the available resources and obtain a cost-effective improvement of health in people living with HIV. Additional research, with the involvement of different centers and the use of patient-specific data, are recommended to consolidate the findings of this analysis.

## Additional file

Additional file 1: **Table S1.** Results of the hospital care expenditure analysis. **Table S2.** Unit costs considered to evaluate ambulatory care expenditure. (DOCX 18 kb)

## Abbreviations

AIDS: Acquired Immune Deficiency Syndrome
ALT: Alanine aminotransferase
AST: Aspartate aminotransferase
ATC: Anatomical Therapeutic Chemical Classification System
cART: Combined Antiretroviral Therapies
CD4 +: CD4+ T Lymphocite
CT: Tomography
DAA: Direct-Acting Antiviral Agents
DH: Day Hospital
DRG: Diagnosis-Related Group
FDC: Fixed-Dose Combination Regimens
HDL: Cholesterol high-density lipoprotein
HIV: Human Immunodeficiency Virus
ICD: International Classification of Diseases
INSTI: Integrase Strand Transfer Inhibitor
IQR: Interquartile Range
LDL: Cholesterol high-density lipoprotein
LOS: Length Of Stay
NHS: National Healthcare System
NNRTI: Non-Nucleoside Reverse Transcriptase Inhibitors
NRTI: Nucleoside And Nucleotide Reverse Transcriptase Inhibitors
PLWHIV: Persons living with human immunodeficiency virus
PI: Protease inhibitors
RNA: Ribonucleic acid
SD: Standard deviation
STR: Single Tablet Regimen
TAF: Tenofovir alafenamide
