# Supplementary material for: How has the cost of antiretroviral therapy changed over the years? A database analysis in Italy
Source: BMC Health Serv Res. 2018 Sep 6;18:691. doi: 10.1186/s12913-018-3507-x (PMC6127985; doi:10.1186/s12913-018-3507-x)
Supplement: Supplementary file 1 — Table S1. Results of the hospital care expenditure analysis. Table S2. Unit costs considered to evaluate ambulatory care expenditure. (DOCX 18 kb) [file 12913_2018_3507_MOESM1_ESM.docx]

Table S1. Results of the hospital care expenditure analysis.

|  | Day hospital | | Hospitalization | |
| --- | --- | --- | --- | --- |
| Year | 2009 | 2015 | 2009 | 2015 |
| Frequency (N) | 93 | 2 | 265 | 129 |
| Weighted cost (€) | 268 | 270 | 5,432 | 5,920 |
| Total estimated expenditure (€) | 24,924 | 540 | 1,439,526 | 763,662 |

Table S2. Unit costs considered to evaluate ambulatory care expenditure.

| Exam/service | Code [23] | Cost (€) |
| --- | --- | --- |
| General visit | 89.7 | 20.66 |
| Collection blood sample | 91.49.2 | 2.58 |
| Blood count | 90.62.2 | 3.17 |
| Alanine aminotransferase (ALT) | 90.04.5 | 1.00 |
| Aspartate aminotransferase (AST) | 90.09.2 | 1.04 |
| Bilirubin | 90.10.5 | 1.41 |
| Calcium | 90.11.4 | 1.13 |
| Cholesterol high-density lipoprotein (HDL) | 90.14.1 | 1.43 |
| Cholesterol low-density lipoprotein (LDL) | 90.14.2 | 0.67 |
| Total cholesterol | 90.14.3 | 1.04 |
| Creatinine | 90.16.3 | 1.13 |
| Alkaline phosphatase | 90.23.5 | 1.04 |
| Phosphor | 90.24.5 | 1.46 |
| [Gamma-Glutamyl Transpeptidase](https://www.urmc.rochester.edu/encyclopedia/content.aspx?contenttypeid=167&contentid=gamma_glutamyl_transpeptidase) | 90.25.5 | 1.13 |
| Glucose | 90.27.1 | 1.17 |
| L-lactate dehydrogenase | 90.29.2 | 1.13 |
| Lipase | 90.30.2 | 2.58 |
| Blood typing | 90.81.5 | 17.09 |
| HIV quantitative RNA analysis | 91.22.3 | 54.23 |
| Total per exam | | 94.43 |

HIV=human immunodeficiency virus; RNA=Ribonucleic acid.
